# Dynamic development of the pancreas from birth to adulthood

**DOI:** 10.3109/03009734.2016.1154906

**Published:** 2016-04-02

**Authors:** Susan Bonner-Weir, Cristina Aguayo-Mazzucato, Gordon C. Weir

**Affiliations:** Joslin Diabetes Center, Harvard Medical School, Boston, MA, USA

**Keywords:** Beta cells, neogenesis, postnatal growth

## Abstract

After birth the endocrine pancreas continues its development, a complex process that involves both the maturation of islet cells and a marked expansion of their numbers. New beta cells are formed both by duplication of pre-existing cells and by new differentiation (neogenesis) across the first postnatal weeks, with the result of beta cells of different stages of maturation even after weaning. Improving our understanding of this period of beta cell expansion could provide valuable therapeutic insights.

The study of pancreatic development has focused for many years on the embryonic specification and differentiation of the various islet cells and the regulation of these processes. However, development of the postnatal endocrine pancreas is also a complex process that involves the maturation of islet cells and marked expansion of their numbers. The work of Claes Hellerstrom and his colleagues provided a strong basis for our knowledge of beta cell expansion and the functional immaturity of fetal/neonatal beta cells. In this paper we will focus on some concepts that stem from this early work with regard to beta cell function, mass, and turnover.

## Variable age and function within the beta cell population

It is important to remember that beta cells change during their lifetime in their capacity for insulin secretion and proliferation. Newborn beta cells have immature secretory function and a high rate of proliferation (1). With time, their secretory capacity is enhanced, particularly for glucose-stimulated insulin secretion (GSIS), and their ability to replicate declines. Then beta cells can progress to senescence with some decline in secretory function, loss of replication, and susceptibility to death. A key point is that the age of beta cells is only partially linked to the age of the person. The slow turnover of beta cells through life results in beta cells of different ages. Thus, a young person will have both newborn and older beta cells, as will an older person; the difference is that the older person can be expected to have a higher proportion of older cells. The changing proportions of new and old cells would influence the overall insulin secretion. This heterogeneity becomes even more complicated when considering that there are two ways to create new beta cells. In spite of some past controversy, it has become clear that neogenesis, the generation of new islets from pancreatic duct cells, takes place throughout life (2–5). These newly formed cells must go through a maturation process. However, new beta cells are also generated by duplication of existing beta cells, and their maturity is likely dependent of the maturity of the parent cell. Thus, although not rigorously studied, when immature cells divide, the daughter cells are expected to be immature, while the daughter cells of older beta cells are probably mature.

## Maturation of insulin secretion

Insulin secretion of immature beta cells is characterized by weak GSIS, with relatively better responses to nutrients such as amino acids (1). Not only do immature beta cells respond poorly to increased glucose levels, but they fail to shut off insulin secretion efficiently when glucose levels are low (6), an important feature of mature beta cells, which provides protection from hypoglycemia.

Exposure of immature beta cells to glucose itself can enhance maturation. Freinkel and Hellerstrom (7) showed that fetal rat islets cultured for only 8 days in RPMI 1640 media with 11 mM glucose compared with controls at 2.8 mM had near-adult levels of secretory responses to a challenge of 16.7 mM glucose. Additionally, we showed that islets from 2-day-old rats (P2) treated for 4 days with either adenovirus expressing the transcription factor MafA or T_3_ approached the secretion of adult cells (8,9). Yet Bliss and Sharp (10) showed with perifused rat islets that glucose-responsive insulin secretion was present by 14 days after birth (P14), but that even at P21 fully developed glucose responsiveness of the adult had not yet been reached. Why does it take so long *in vivo* to obtain full GSIS? The best explanation seems to be that large numbers of beta cells are generated late in the first postnatal month and that their secretory immaturity obscures the robust secretion from a more mature population of cells.

To understand the molecular basis of this immaturity several studies identified single genes with low expression thought to play a role in the maturation process. To examine a larger number of genes we performed microarray analysis of laser-captured beta cells from P1 rats compared to those from adults and found that many genes were differentially expressed, including many that were important components of specialized NADH mitochondrial shuttles that are unique to beta cells (11). Some of these genes with low expression at P1 (e.g. pyruvate carboxylase, maleic enzyme1, maleate dehydrogenase, glutamate oxaloacetate transaminase, and glycerol phosphate dehydrogenase 2) only reached adult levels of mRNA by P28, with sharp increases only after P21. This suggests that important mitochondrial shuttles are undeveloped, most notably the glycerol–phosphate, malate–aspartate, pyruvate–citrate, and pyruvate–malate shuttles. Thus, the metabolic specialization found in adult beta cells for amplifying the ATP derived from glycolysis is deficient in neonatal beta cells. Other key beta cell genes had low expression and only reach adult levels by P28; these include insulin, glut 2, PCSK1, and GLP1R (8).

## Changes in beta cell mass during first postnatal month

**Figure 1.**
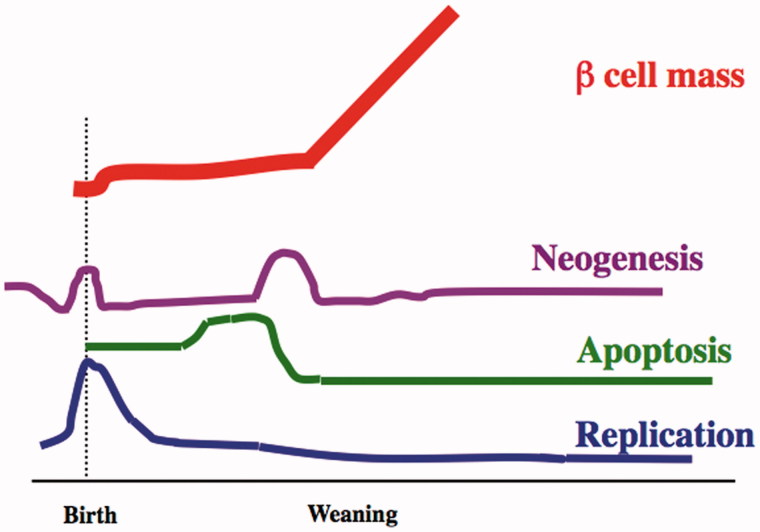
Schematic drawing of the dynamic changes in the determinants of beta cell growth from the first postnatal month into adulthood. This diagram (drawn not to scale) is based on the data in Scaglia et al. (12). Extending past this same period is the process of functional maturation of the beta cells.

The first postnatal month is a time of many changes for the endocrine pancreas (Figure 1) as documented by a longitudinal study of the determinants of the beta cell mass over the first postnatal month (P2, 9, 13, 17, 29, 24, and 31) in Sprague Dawley rats (12). Replication was highest perinatally, with a gradual decline over the month. At P2, 6 h BrdU incorporation labeled 4.7% ± 0.3% of beta cells; by P13 it had fallen to 2.2% ± 0.5% and then to 1.8% ± 0.2% by P31. This is still greater than the lower level seen in 2–3-month-old adults (0.22% ± 0.06%). Similarly, throughout this month the frequency of apoptotic beta cells was higher than in the adult (0.5%), being 1.54% ± 0.22% at P2, P9, and again after 20 days. However, there is a very interesting increase of apoptosis (3.64% ± 0.45%) between P13 and P17 of age, which precedes weaning. This wave of apoptosis has attracted the interest of immunologists trying to understand the pathogenesis of type 1 diabetes because of the release of antigens. In spite of this increased apoptosis, beta cell mass was stable from 2 to 20 days (range: 0.91 ± 0.2 mg to 1.33 ± 0.23 mg) and increased thereafter. To maintain the constant beta cell mass in the face of decreased replication and increased incidence of apoptosis in the beta cells a wave of neogenesis of beta cells is strongly suggested. We estimated the cell number based on these data and determined that replication could only account for 50%–70% of the beta cells present at P31 (13).

## Evidence of neogenesis within the first postnatal month

Most evidence for neogenesis has been correlative, being indicated by insulin-positive cells embedded in ducts, co-staining for cytokeratin and insulin, or by increased presence of small clusters of islet cells in situations of increased beta cell mass such as pregnancy. More convincing support for the presence of neogenesis can be provided by lineage tracing in mice. Data from three of our experimental models provide further support for the estimate that neogenesis accounts for 30%–50% of the new beta cells found during the first postnatal month, with further support provided by other groups.

### Transient expression of MMP2, marker of newly formed beta cells

Using gene expression data from laser-captured microdissected beta cells of P1 rats, we searched for markers of newly formed beta cells using immunostaining (14). One of these markers, MMP2 protein, had strong staining in beta cells at embryonic age E20 that was even more intense at birth. After 48 hours, MMP2 expression started to decrease and had disappeared by P7. However, at P17, a time predicted to have more neogenesis, 51% of the islets (545/1,074; from 4 rats) had MMP2 co-staining with insulin, and the pattern was lobular (Toschi et al. unpublished data). This expression was transient, with 16% at P14 (127/798 islets; *n* = 2) of the islets having MMP2 immunostaining, 17% (198/1159; *n* = 4) at P20, and only 1% (10/1,096; from 4 rats) at P24. Essentially no positive staining was found in adult rats.

### Lineage tracing using carbonic anhydrase II Cre:ROSA26-LSLβGal mice

Carbonic anhydrase II (CAII) only starts to be expressed in pancreatic ducts at the very end of gestation and has been considered a duct-specific marker within the pancreas (15). Using Cre-lox lineage tracing driven by the CAII promoter, we found that at 4 weeks after birth both islets and acini were marked as having been formed from cells that once expressed CAII; 38% of the islets were marked (17% of all insulin-positive cells) as well as a number of acinar cells, with some lobes being strongly marked and others not (16).

### Duct-specific deletion of Pdx1

Using these CAII^Cre^ mice crossed with Pdx1^floxE2^ mice (17) so that *Pdx1* expression would be specifically deleted from ducts only starting around birth, we found *Pdx1* is not necessary for formation of new beta cells from postnatal pancreatic ducts; however, it is required for these newly formed beta cells to become fully mature (18). What was striking was that there were three types of islets in each section of adult pancreas: 30% with normal strong PDX1 expression, 20% with little to no PDX1 expression, and 50% mixed insulin-positive cells with and without PDX1 staining. We interpret the group with strong PDX1 as those islets formed before birth, and those without PDX1 as being formed by neogenesis after birth. The group with mixed expression suggests a coalescence of newly formed PDX1 beta cells with islets or clusters of beta cells formed before birth. Alternatively, these cells could have arisen from the intra-islet ducts shown in a recent study from the Gittes lab (3). The PDX1-deficient beta cells accounted for 32% of all beta cells in 10-week-old mice; the lineage of these cells from ducts was verified by their expression of the recombination reporter eYFP.

### Other studies finding increased islet number in the early postnatal period

In addition to our studies, several other groups have provided documentation of a substantially increased number of islets or insulin-positive cell clusters occurring from birth to 2 months of age. Chintinne et al. concluded that about 30,000 beta cell clusters of less than 50 μm formed between P2 and 10 weeks in Wistar rats (19). In mice Peng et al. (20) reported about 50 islets per pancreas at 1 week but 900 at 2 months, after which time it was stable. With MIP:GFP mice Jo et al. (21) modeled that there was fission of larger islets after birth and that within the first 3 weeks postnatally 800 new islets were formed: 300 by fission and 500 by neogenesis. There is the caveat for such enumeration of islets that small clusters initially below the limit of detection may enlarge by replication and be erroneously counted as being newly formed by neogenesis. Realizing this, the above-mentioned studies had lower detection limits of one (19), two (20), or four (21) cells.

## Neglected issue in postnatal pancreas development: rapid pancreatic growth from 2 to 4 weeks of age

**Figure 2.**
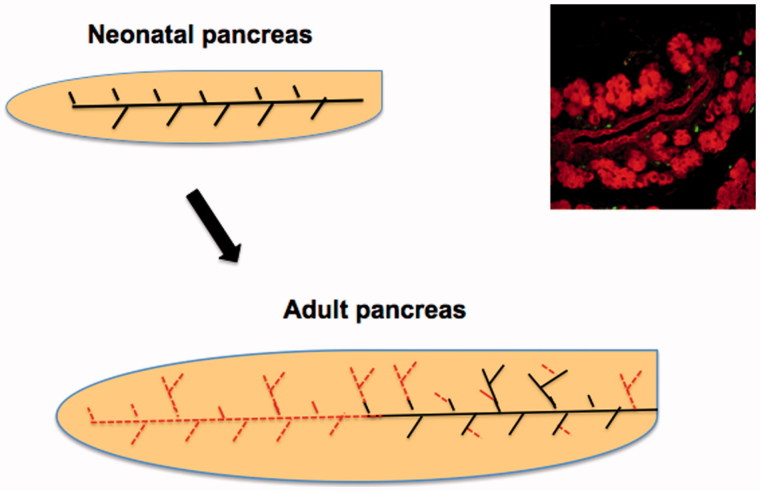
Pancreatic growth is by formation of new lobes and further branching of ducts to form new acini and not by continued growth by adding cells to individual acini. The dotted (grey or red) lines in the adult pancreas represent those ducts that grew after birth as the pancreas has extensive growth.

In the first postnatal month there is marked growth of the mouse or rat accompanied by even more growth of the pancreas. Between P2 and P9 in rats both the body weight and the pancreatic weight have 1.8-fold increases, but between P13 and P31 the body weight increases 2.6-fold whereas the pancreatic weights increase 5-fold (12). A similar divergence in mice is seen with a 2.6-fold increase in body weight and 3.7-fold in pancreatic weight from 2 to 4 weeks of age (18). In the first week there is extensive growth of the exocrine pancreas, with formation of new acini such that the loose appearance of the sectioned pancreas seen at birth is lost as the pancreas develops the packed appearance as seen in the adult. The subsequent growth cannot be accounted for by enlarged acini from replication but rather as extensions of the pre-existing lobes of pancreas as well as formation of new lobes by proliferation of the ducts accompanied by their subsequent differentiation into islets and acini (Figure 2). With extension of the main ducts and branching formation of new lobes, as we saw in the regeneration model of partial pancreatectomy (22,23), the distribution of islets eventually is similar in lobes that developed at different times, each with larger islets closer to the main ducts and smaller islets towards the periphery. The marking of lobes of pancreas in the lineage-tracing experiments (above) with CAII^cre^:ROSA26 LSL mice supports the concept of new lobe formation within the first neonatal month.

The time between 2 and 4 weeks of age is one of changing diet and systemic hormonal changes as rats start adding chow to their milk diet (24). The rapid growth of pancreas between 2 and 4 weeks and the estimates of new islets and of newly formed beta cells support the concept of a substantial wave of neogenesis occurring just before weaning. While it is unclear how long the maturation process normally takes for individual beta cells, glucose-responsive insulin secretion was seen by P14 (10) and became more robust by P21 but still failed to reach the even greater secretory responses seen in adults (2–3 months old). Our premise is that the continued presence of immature beta cells even as late as P21 accounts for this difference.
